# Life and Death

**Published:** 1877-08

**Authors:** 


**LIFE AND DEATH.**—One half the human family dies under seventeen years of age. Nine tenths of all who are born ought to complete their "three-score years and ten," because nine tenths of all diseases are avoidable by the steady practice of temperance and such out-door activities as are encouragingly remunerative. There is a still more specific method of lengthening life in healthfulness and vigor, and one which is practicable by the masses. Colds or constipation immediately precede or attend almost every case of ordinary disease. The latter can be antagonized by abstinence, cleanliness, and warmth for thirty-six hours; and a cold need not be taken once a year if three things are attended to. Avoid chilliness, damp clothing, and cooling off too soon after exercise.

